# Design considerations for photovoltaic energy harvesting in wearable devices

**DOI:** 10.1038/s41598-022-22232-x

**Published:** 2022-10-28

**Authors:** Katherine A. Kim, F. Selin Bagci, Kristen L. Dorsey

**Affiliations:** 1 Department of Electrical Engineering, National Taiwan University, Taipei, 106 Taiwan; 2 Department of Electrical and Computer Engineering, Northeastern University, Boston, MA 02115 USA

**Keywords:** Electrical and electronic engineering, Photovoltaics

## Abstract

Wearable technology is emerging as a solution for various bio-mechanic and health-metric applications. Solar photovoltaic energy is a viable supplemental power source that can reduce battery size requirements in wearables. This study outlines the considerations for a wearable sleeve device and its associated power converter system using commercially-available flexible photovoltaic panels located on the forearm. Investigation of the effects of curvature shows that while curvature of the panel around a forearm does reduce output power, the angle relative to the light source has a more pronounced effect on both output power and voltage characteristics. Among various panel arrangements on the forearm, that with five individual panels of smaller width provided the highest output power after the boost converter power stage. Testing under various static positions, the PV sleeve provided up to 94 mW outdoors, which can effectively reduce the battery size while maintaining user safety.

## Introduction

Innovations in sensing, computing, and fabrication have moved the capabilities of wearable devices beyond biometric monitoring (e.g., heart rate^1^, step count^2,3^) into interrogating more complex bio-mechanics and health metrics (e.g., activity classification^4^, ergonomic monitoring, gesture recognition^5^). As the monitoring and analysis of physiological signals and body motions becomes more sophisticated, so will the demands for computation and power. Supplying sufficient power to such devices, without requiring large batteries or frequent recharge cycles, is a challenge that is likely to grow in importance as wearable devices become more ubiquitous. One promising approach to extending available power is by supplementing battery capacity with energy scavenged from a user, their motions, or the environment.

Many potential energy sources are available for harvesting in wearable contexts^6–10^, including solar, body motions, radio waves, and thermal gradients between skin and ambient air. Each of these energy sources carry strengths and drawbacks depending on the context(s) for use. Body motions can be harvested using piezoelectric^11^, electromagnetic^12^, or triboelectric^13^ generators but require mechanical motion (e.g., vibrations from walking) and therefore a physically active user. Power density for energy harvested from human motion is estimated to be around 4 $\mu$Wcm$^{-2}$^14^. Thermal gradients between skin and the surrounding air can be harvested using thermoelectric generators, with a typical power output on the order of 10 $\mu$Wcm$^{-2}$^15^. However, the output decreases with smaller thermal gradients between skin and ambient temperatures, such as in a warm room or if the user has low skin temperature or poor circulation. Ambient electromagnetic harvesters can extract radio frequency energy, but the scavenged power depends on distance from radio frequency sources^16^, with a power density^6^ on the order of 1 $\mu$Wcm$^{-2}$ . In contrast, the power that photovoltaic (PV) cells can supply is independent of user activity, with reported power densities of 10-100 $\mu$Wcm$^{-2}$ under ambient indoor light and 100 mWcm$^{-2}$ in direct sunlight outdoors^6^. Due to their relatively large power production, we will focus on power output from PV cells in this work.

Several user studies^17,18^ have shown that users prioritize form factor, functionality, and battery life in selecting wearable devices. Successful integration of solar energy harvesting into garments, therefore, requires a combination of flexibility, high performance, and efficiency. Additionally, since these garments may be made at large processing scale, processes that deliver commercially-available panels are of greatest consideration to wearable devices over the next five years. PV cell types that show promise for wearable applications include textile-based PV cells^19–21^ and flat-surface PV cells. In general, fiber-based PV cells^22–25^ use fibrous materials (e.g. metal, optical, or conductive strands) that are woven into larger structures^22^. Fiber-based PV cells offer two advantages: 1) the textured form of fibrous cells can lead to increased absorption of scattered light; and fiber-based PVs have closer (but not exact^26^) properties to textiles than panels fabricated with a continuous, planar substrate. Recent work in another fiber-based platform^27^, “solar E-yarns”, preserves the mechanical properties of textiles while maintaining the efficiency for which crystalline-silicon (c-Si) PV cells are known, by encapsulating miniature solar cells within yarn. A third approach in textile-based PV cells uses spray-coated fabrics^21^ to form the energy harvesting layer.

In contrast to fiber-based PV cells, flat-surface flexible PV cells^28^ are inorganic, organic, or hybrid solar cells that are fabricated on mechanically compliant substrates. Recent advances in scalable fabrication have made flexible, silicon-based thin-film PV cells commercially available^29^. While these panels are highly flexible and can be machine sewn onto fabric^30^, they lack the appearance or mechanical properties of textiles. The viability of these cells have been demonstrated in a range of wearable applications^31–33^. While recent and ongoing work in developing fiber-based PV cells offers significant promise for future applications in wearable devices, the current challenges in generating sufficient power and additional development required for them to become commercially available suggest that near-term applications should investigate wearable devices designed with flat-surface flexible solar cells.

Previous works have demonstrated full garments with PV cells, including a jacket^34^ and a wristband^35^. PV cells in garments have a few critical design challenges. One challenge is predicting the effects of deformation and self-shading from body poses on the panel’s output. A previous study^36^ addressed the effects of bending on amorphous silicon (a-Si) panel output and found that bent panels produced less power and had a higher maximum power point (MPP) voltage. However, the translation of these findings to wearable contexts and body poses is less clear. A second challenge is optimizing the size, orientation, and arrangement of the PV panels to maximize power production within the size of the garment. The final challenge is estimating the power produced and converted from wearable PVs under realistic body positions. Some realistic harvested energy capacity of flexible PV panels over a period was explored^37^ previously but without addressing realistic body positions.

In this work, we discuss panel, power converter, and garment design choices that are critical to efficient power conversion in wearable device contexts. We also characterize the power outputs of flexible, commercially-available, a-Si panels under the load conditions expected in wearable devices. Finally, we measure power generated by a PV garment prototype in order to estimate the potential of flexible PV panels to supplement battery capacity in wearable applications.

## Design considerations

The selection of PV panel type presents two important characteristics: the efficiency in converting light intensity into electrical power, and the mechanical flexibility and durability of the panel. Common flexible, thin-film PV materials include copper indium gallium selenide (CIGS), cadmium telluride (CdTe), and hydrogenated amorphous silicon (a-Si:H). Reported flexible polymer foil panel efficiencies are dependent on the material selection, as CIGS can reach 20.4%, CdTe can reach 13.8%, and a-Si:H can reach 7.1%^38^. Amorphous Si panels have previously been demonstrated in contexts such as continuous monitoring of blood glucose from sweat^33^. Its amorphous structure also helps reduce damage (and consequent reduction in power output) during flexion^38^. In this work, we discuss design considerations related to a-Si:H (hereafter referred to simply as a-Si) PV cells mounted on flexible plastic.

### DC-DC converter and control

The interface between PV panel and wearable device is another important design choice. The PV cells are connected to form a panel and the electrical connections within the panel (i.e., series, parallel, or a series-parallel network) directly affect the panel’s output characteristics. Loads within the wearable device (e.g., sensors, microcontroller, battery, displays) can be connected directly to a panel when the load’s voltage rating is within the output voltage range of the panel. However, because the load characteristics (i.e., impedance) are not optimized for power transfer, the PV panel may not operate at the point that allows it to maximize power output. To supply loads with the largest power possible, a converter is used to control the operating voltage of the PV panel and either step the voltage up or down to meet the demands of the load. A switched-mode power supply, such as a buck or boost converter, is typically chosen due to its high conversion efficiency and straight-forward implementation.

For highest power transfer and efficiency, the power converter must regulate the PV source voltage at the MPP (i.e., the voltage at which output power is maximized). There are various maximum power point tracking (MPPT) algorithms that track the MPP and can be directly implemented in the standard DC-DC converter topologies^39^. One of the most-widely used algorithms in PV applications is the perturb and observe (P &O) algorithm, which actively steps the operating point (perturb) and measures the resulting power change (observe) to continually move towards the MPP. In this way, it continuously tracks the maximum of the PV power curve and can be implemented on a low-cost microcontroller. In comparison to other algorithms such as incremental conductance, ripple correlation control, or intelligence-based techniques, P &O does not require calibration, is not computationally intensive, and the circuitry is inexpensive to implement^40^, so it strikes a balance between power maximization and ease of implementation.

**Figure 1:**
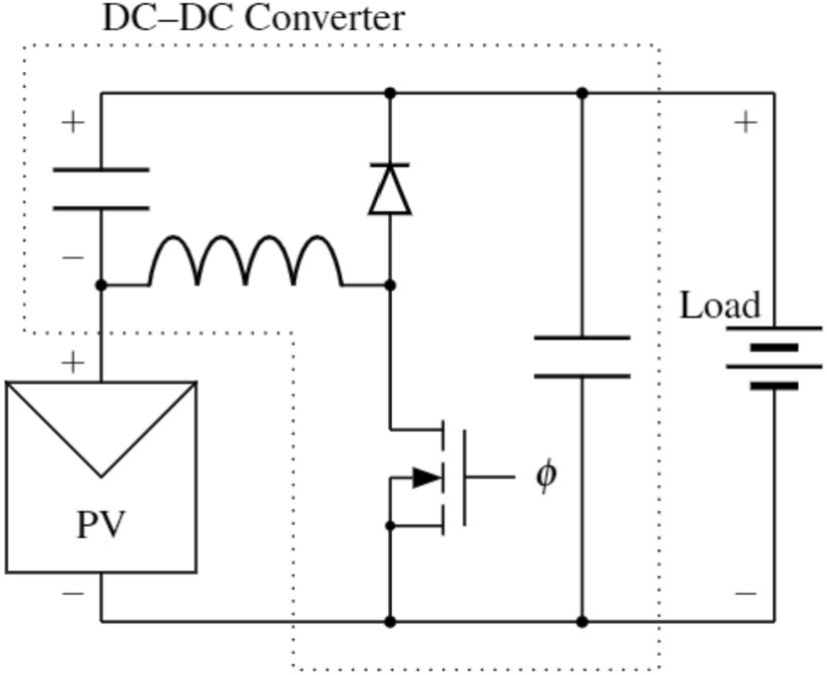
A diagram of the PV energy harvesting system with boost converter, where the gate of the transistor is driven by a gate driver circuit ($\phi$) and controlled by a microcontroller.

Given a combination of battery and PV panel, the PV MPP voltage may be above or below the battery’s load voltage. The cells within the panel may be connected in series, where each cell within the panel shares the same current, or in parallel, where each cell shares the same voltage. Lee et al.^41^ identified that parallel-connected PV panels, with a lower MPP voltage than series-connected panels, result in higher output power for wearable PV panels in conditions such as wearable devices where nonuniform lighting across the panel is expected. Hence, selecting a parallel-connected PV with a lower MPP voltage and a boost converter topology (Fig. 1) to step up the voltage is an effective design to maximize PV power harvest over a range of lighting scenarios. While the voltage bus at the converter output powers all loads within the device (e.g., sensors, microcontroller), it is dominated by the characteristics of the battery. The operation of the boost converter is controlled by a low-power microcontroller, which adjusts the pulse-width modulation (PWM) signal used to drive the MOSFET based on the MPPT control algorithm.

**Figure 2:**
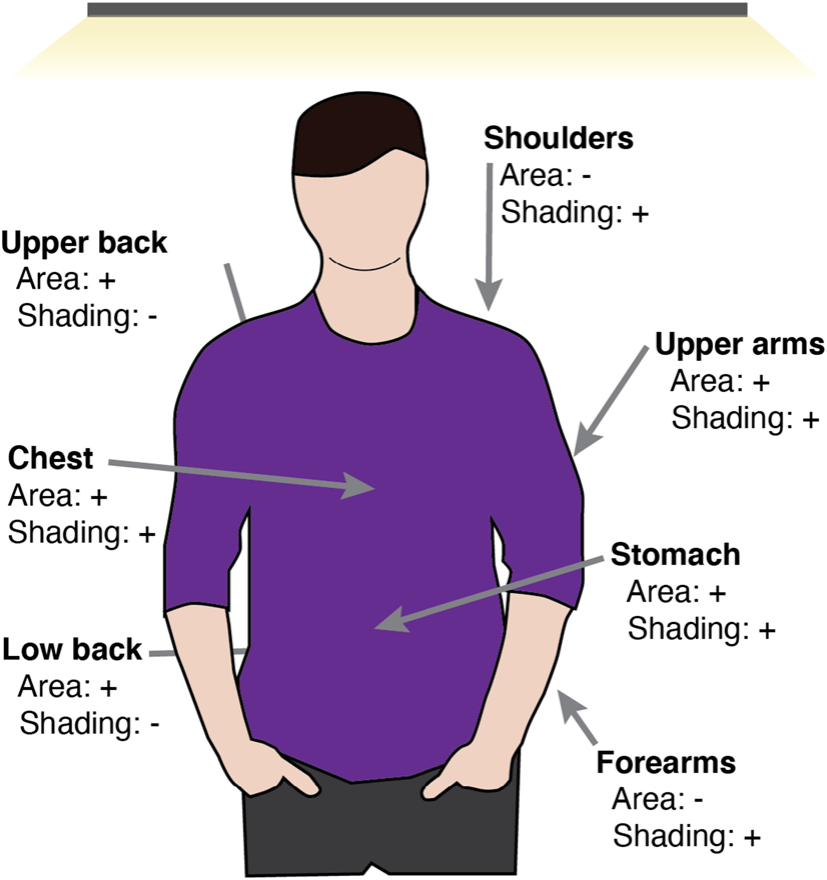
Potential flexible PV panel locations on the body. Placement on each location has strengths and drawbacks related to surface area and potential shading during normal motions.

### Battery capacity and panel sizing

To design the power system architecture for a PV-powered wearable device, the average current consumption of the device load, average power $P_{avg}$, and anticipated battery capacity should first be identified. These considerations are illustrated through a hypothetical design example of a wearable device with a 4-MHz microcontroller (MCU), a 9-axis inertial measurement unit (IMU), eight strain sensors, a Bluetooth MCU module, and one display light (LED). If the load voltage is 3.3 V and the components are active approximately 50% of the time and otherwise placed in a low-power or sleep mode, the combined average current draw would be around 9 mA. With a 10% margin to account for various losses or leakage, the average current draw will be approximately 10 mA. The average current draw of each component is shown in Table 1.

**Table 1:** Potential loads within a wearable device.

Device	Function	Current
ATmega328	MCU	1 mA
Bluefruit	Bluetooth MCU module	1.9 mA
9-axis IMU	All sensors	2 mA
Strain sensor	Sensing	0.3 mA
LED	Display	1.5 mA

Once the expected load current is identified, the battery capacity can be determined. In a wearable device without energy harvesting, the single-charge usable battery capacity must be higher than 80 mAh to last beyond 8 hours. The battery capacity can be decreased, and energy consumption offset, if solar power is available to re-charge the battery during device operation. Such reduction in battery capacity enables a lighter and lower-profile wearable device.

While reducing battery capacity through solar offers improved wearability, it also presents a trade-off with charging time and temperature rise. A battery’s C-rate is a normalized value based on its nameplate capacity^42^. One study showed that charging a battery at 1.5C (i.e, 1.5 times its nameplate capacity) increased battery temperature by 6 °C, while a rate of 0.5C limited the temperature rise below 2 °C^43^. Since the battery will be worn close to the body, limiting temperature below to 44 °C^44^ (for safety), and ideally 40 °C (for comfort), is critical. The nominal charge rate should be between 0.5C and 1C^42^.

Indoors, PV panels will generate a low nominal power, suggesting that panel area should be as large as possible. However, the supplied current from the panels must remain below 1C, even if the panel is exposed to full sun. The panel power sizing should then be as large as the wearable form factor permits without exceeding the current expected in full sunlight.

### Panel placement

PV panel placement within a garment requires careful consideration of potential deformations imposed on the panel during normal use, the potential for the panels to impede user motion, the area available to place a panel, and the effects of self-shading due to body position and proximity to objects. We evaluate PV panel placement using two metrics: (1) the area available to place the panels on the body, and (2) the potential for self-shading during typical daily activities. Figure 2 is a diagram of these potential locations with their area classifications and potential for shading. The body area metric is taken for the 50-th percentile of people in the United States^45–47^, where low (−) area is defined as less than 500 cm$^2$ and high (+) area is more than 500 cm$^2$. The shading metric is assessed by the presence of nearby objects (e.g., furniture) or self-shading during normal body poses (e.g., sitting, standing), where low (−) is obscured during sitting and high (+) is not obscured.

**Figure 3:**
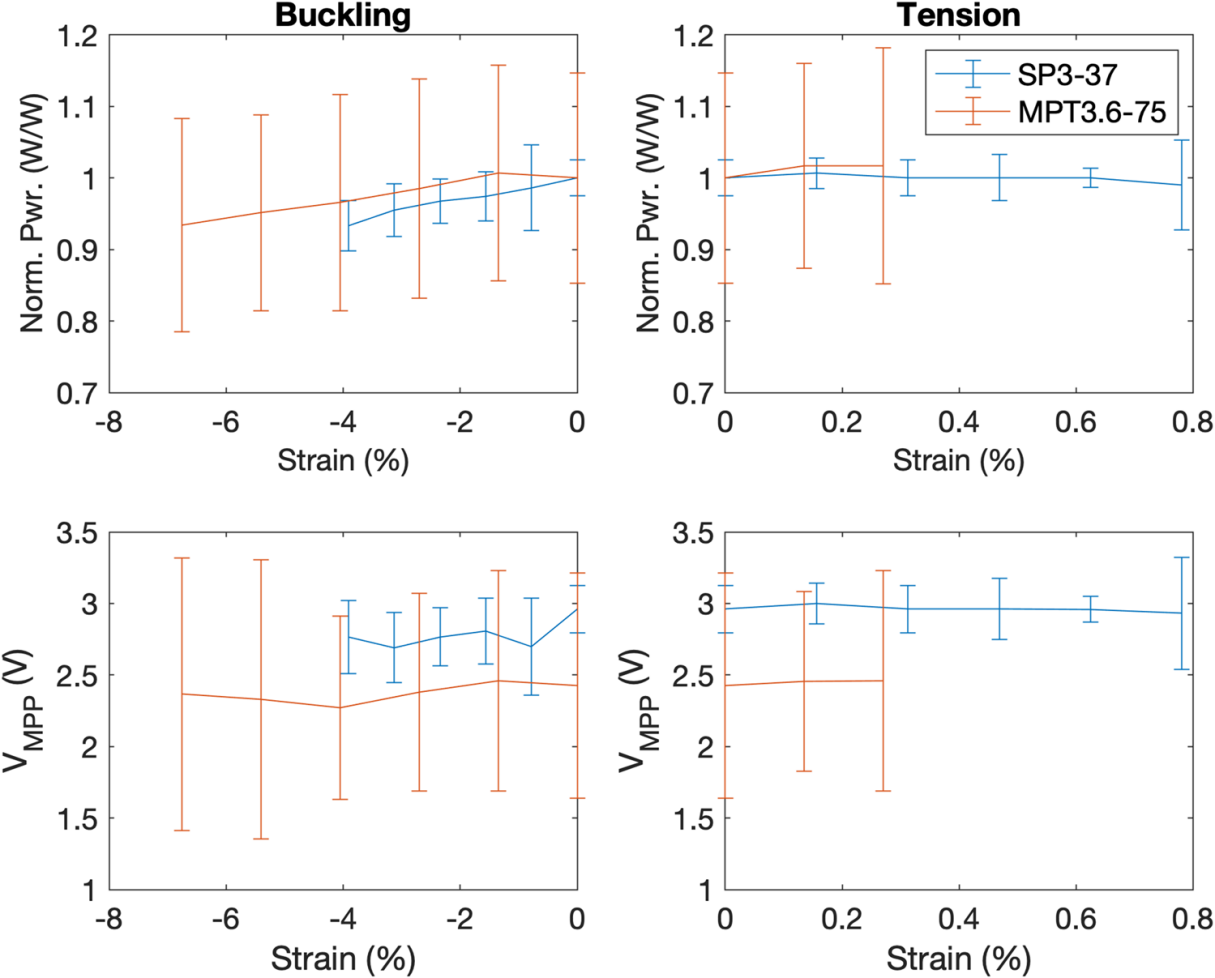
Power generated from panel during buckling (left) and tension (right), normalized to the unstrained output power.

**Figure 4:**
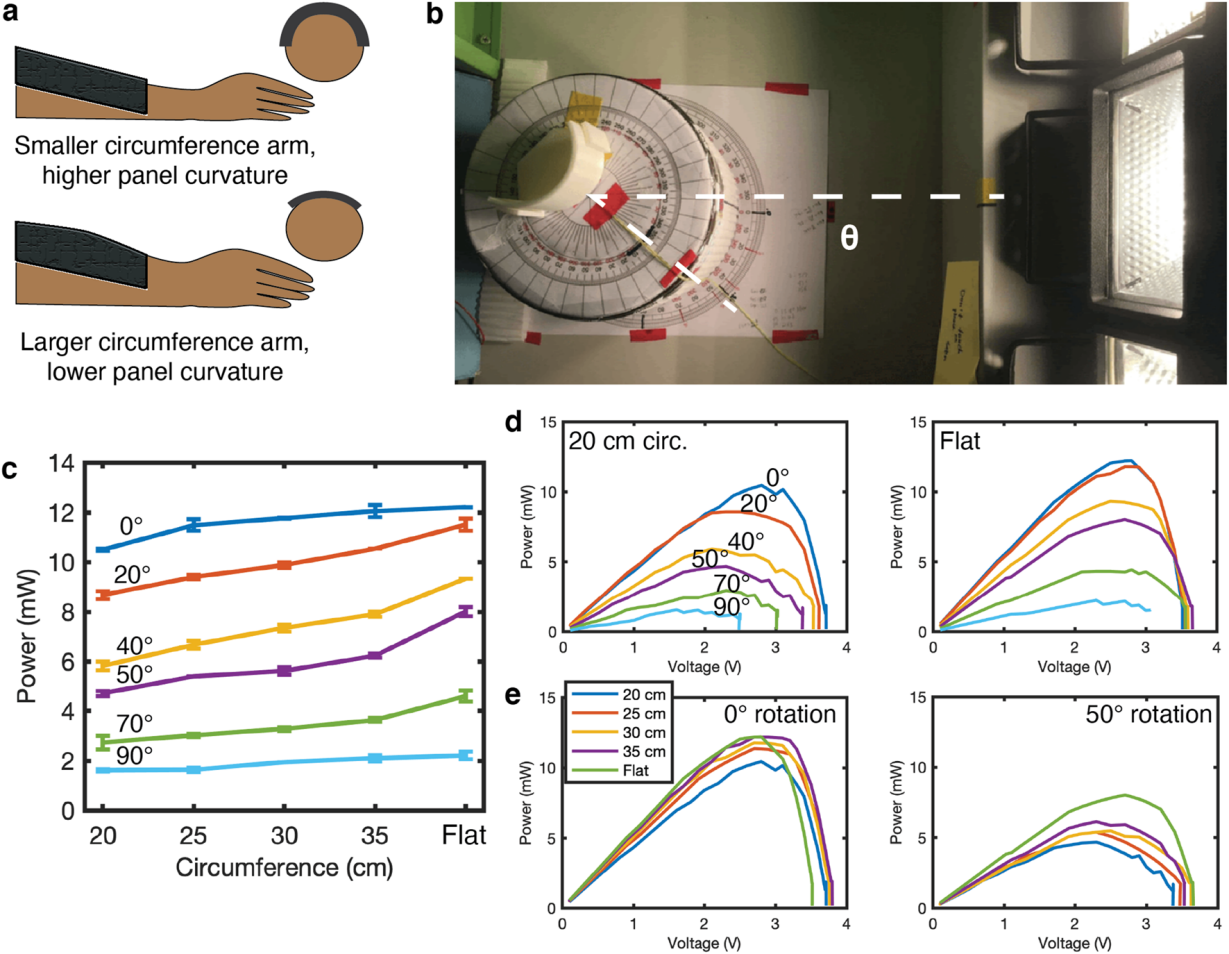
The effect of arm curvature on panel position. (**a**) A diagram of PV panel conformation to large and small circumference arms from the side and cross-section views. (**b**) A photograph of the curvature test fixture, panel, and halogen lights, with a rotation of $\theta$ = 45°. (**c**) The maximum power generated as the rotation and circumference change. While both curvature and angle affect the power generated by the panels, the angle dominates the generated power. (**d**) P-V curves for the 20 cm (left) and flat (right) panels. Decreasing curvature increases maximum power from 12 to 14 mW. (**e**) P-V curves for a 0° angle (left) and a 50° rotation (right). Increasing the angle decreases maximum generated power from 14 mW to 8 mW.

While the upper back and lower back offer large panel areas, these sites are poorly suited for energy harvesting because seated users will cover the panel when seated in a chair. Placement on the upper back and shoulders would require careful panel design and placement to not restrict overhead motions from the shoulders. The stomach and chest, similarly, undergo large expansion during respiration, so panel placement here might restrict users’ natural respiration. In contrast, the upper and lower arms do not bend during activity, which reduces concerns about panel deformation, and are typically not shaded during sitting or standing. The upper arm does have a large rotation to overhead light (and therefore would generate low power). While the forearms offer relatively small panel area when compared to other sites on the torso, the low likelihood of shading and motion restriction make it a useful location for flexible panels. This work focuses on forearm-based PV cell placement.

## Results

### Effects of deformation on panel output

Wearers will impose mechanical stress and deformation, including normal force, tension, and curvature, on the panels during use. We investigate the effect of deformation on the panel output, both in tension and in curvature. Three models of flexible substrate a-Si panels (PowerFilm Solar, Inc.) with different properties were used in the study. Table 2 shows the panel lengths and widths in cm, open circuit and MPP voltages, and expected power (in mW) at an irradiance of 1000 W/m$^2$ (i.e., full sun) and 25 °C.

The MPP voltage and power output of panels under tensile load, normalized to a zero-stress condition, are shown in Fig. 3. Experimental details are available in the “Methods” section. For each panel type, the power increases by less than 10% as the panels are moved from buckling to tension, likely because the panel is not self-shaded once it is not buckled. In tension, the normalized power is stable around its initial value to within 5% until the stop condition is reached. The panel-to-panel variations in MPP voltage variation are large for both panel types, but neither buckling nor tension create a monotonic trend on MPP voltage.

**Figure 5:**
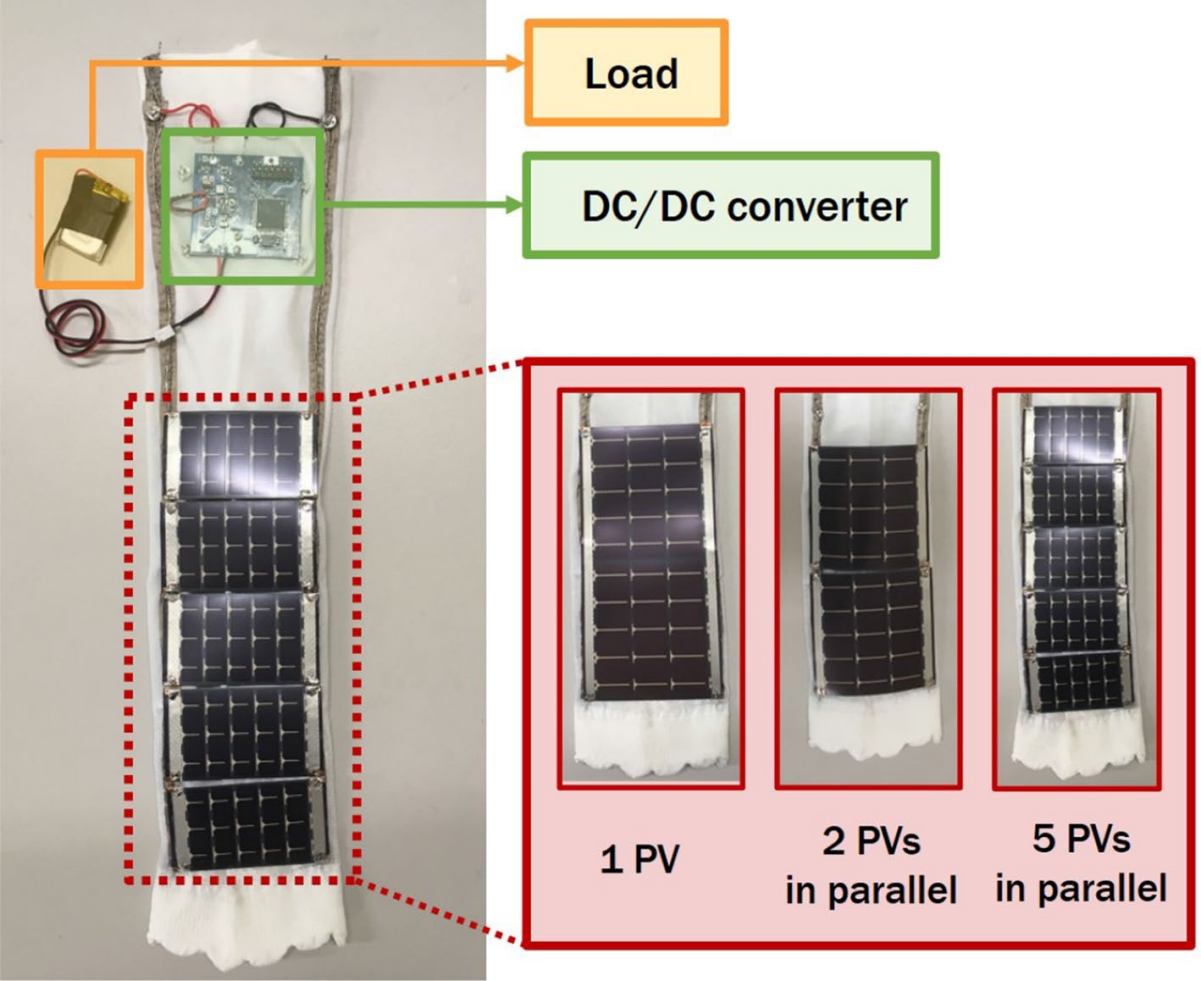
A photograph of the sleeve with a battery load, dc-dc converter, and PV panels covering the forearm. The panel dimensions determine the placement on the sleeve.

**Figure 6:**
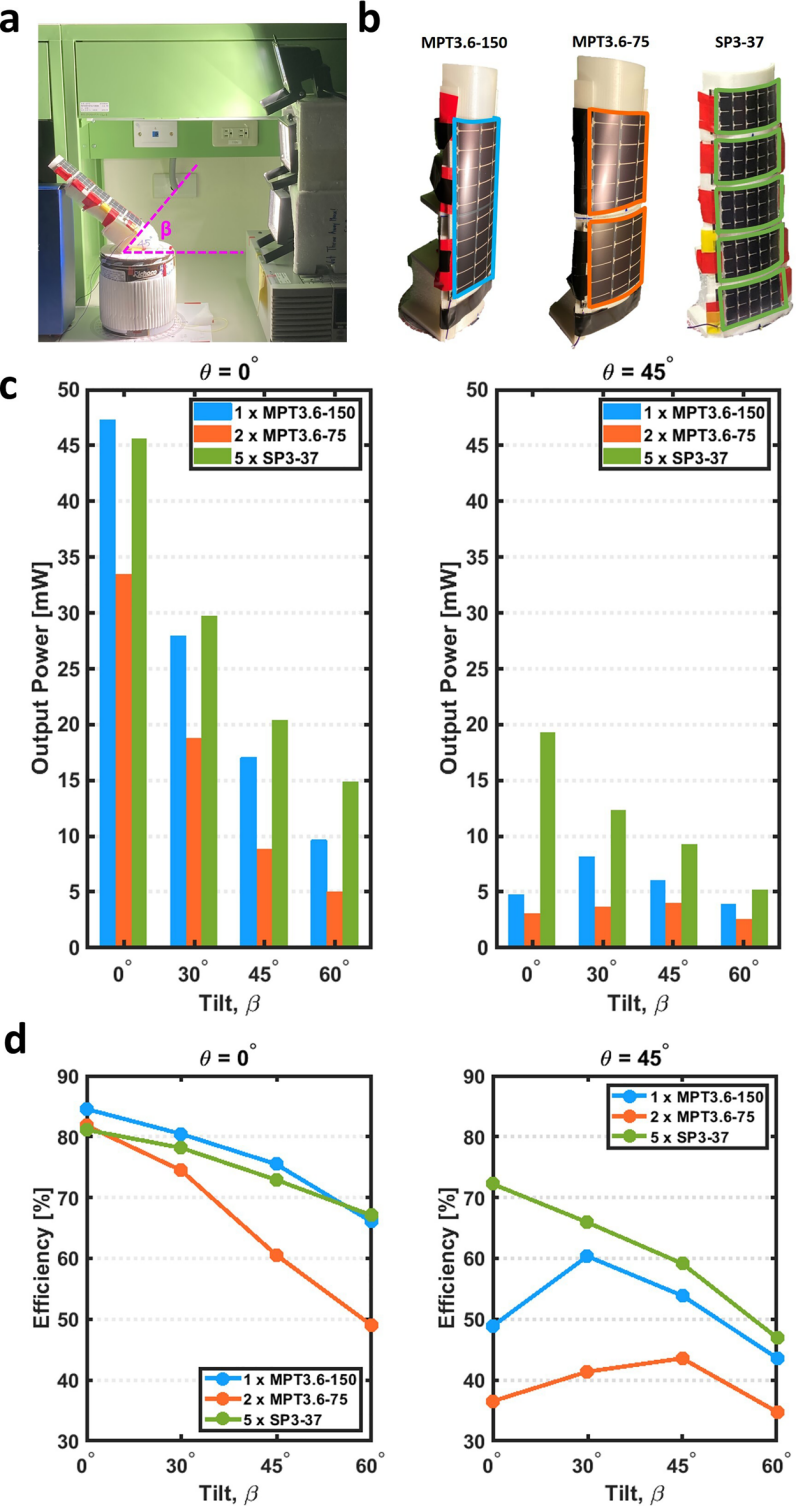
The effect of rotation and tilt on output power. (**a**) A photograph of the forearm test fixture, sleeve, and halogen lights, with tilt angle of $\beta$ = 45$^{\circ }$. (**b**) A photograph of the single-, double-, and five-panel arrangements on the sleeve. (**c**) The output power generated as the tilt angle changes at rotational angles of 0° (left) and a 45° rotation (right). (**d**) Power efficiency as the tilt angle changes at rotational angles of 0° (left) and a 45° rotation (right).

Placing a PV panel on a forearm induces curvature, and wearers with smaller forearms will impose larger curvature than larger forearms (Fig. 4a). Figure 4c shows the maximum power produced as panel curvature and the rotation are increased. The output power is at a maximum (12 mW) for a flat panel perpendicular to the light source and decreases as either curvature or the angle is increased. When the panel is oriented 90° to the lamp, approximately 2 mW of power is still produced. The angle has a more drastic impact on produced power than curvature: increasing the angle from 0 ° to 90 ° cuts power output by a factor of six, while increasing curvature from 0.18 cm$^{-1}$ (35 cm circumference) to 0.31 cm$^{-1}$ (20 cm circumference) decreases output by a maximum of 20%.

**Figure 7:**
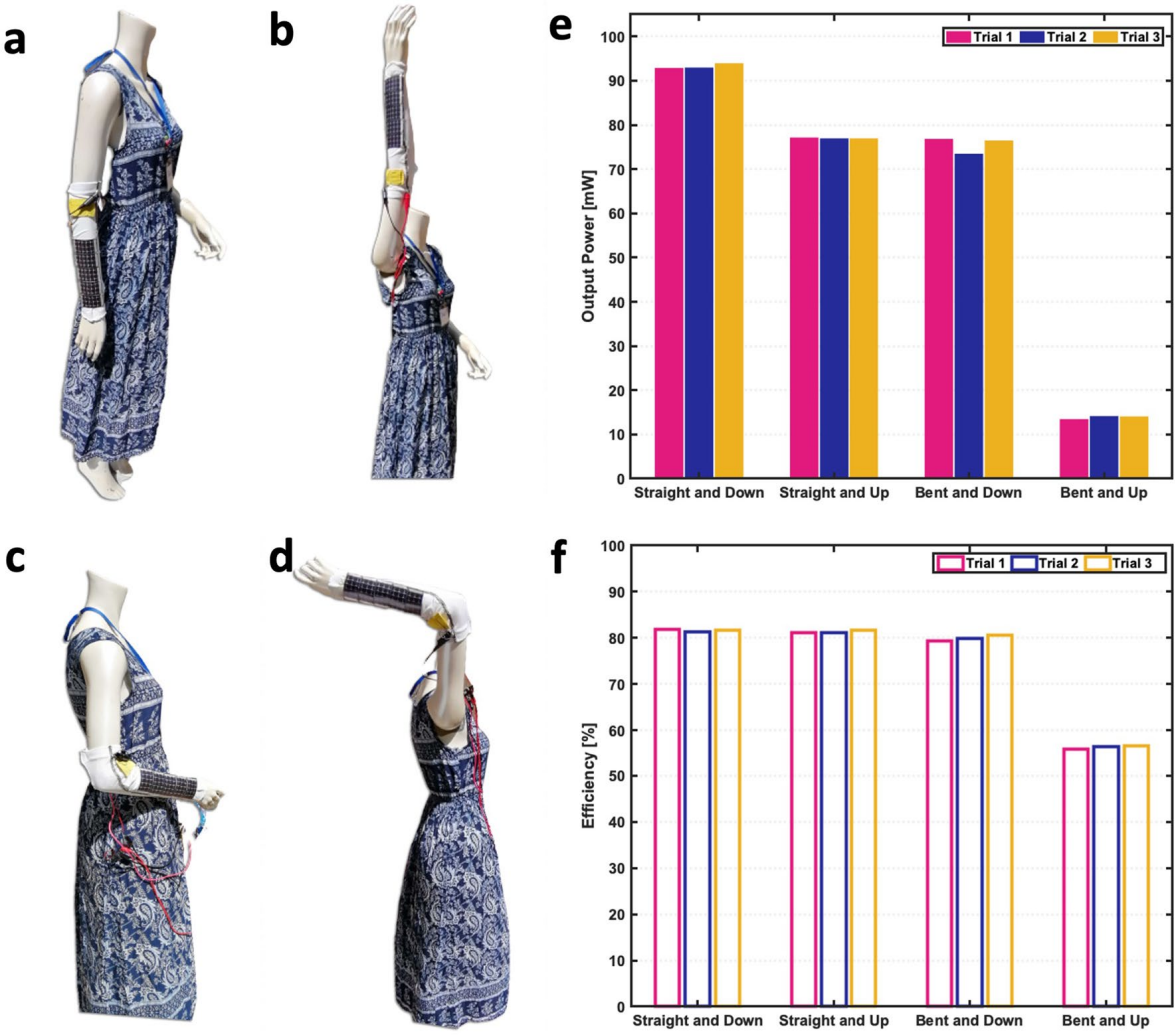
Power measurements from sleeve during different scenarios. The mannequin arm positioned (**a**) down and straight, (**b**) up and straight, (**c**) down and bent, and (**d**) up and bent. (**e**) The average output power over 30 seconds for three trials and (**f**) corresponding efficiency obtained using the five-panel sleeve and converter system during each scenario.

Figure 4d shows the power versus voltage curves with rotation angle for two curvatures: the smallest circumference of 20 cm and a flat panel. As the angle increases, the voltage of the peak point in the curve, which indicates the MPP, decreases for the smaller circumference but the effect is less pronounced for the flat panel. Figure 4e shows the power versus voltage curves with circumferences for a rotation angle of 0 ° and 50 °. When the panel is rotated at 50°, decreasing the circumference has an observable decrease in MPP voltage (peak point of the curve) while the change in MPP voltage is not significant at 0°. These results indicate that for higher curvature (smaller circumference), the MPP voltage will vary as the rotation angle changes while wearing the sleeve.

### PV panel arrangement on sleeve

Another consideration in PV power generation is the practical limits imposed by the garment. For a given forearm size, we want to investigate which configuration of PV panels will yield the greatest power under a range of conditions. The panels are integrated into a sleeve (Fig. 5) and connected in parallel to each other (the anode is oriented towards the outside of the arm and the cathode is oriented towards the inner arm), which enables minimum wiring connections between panels. When the arm circumference increases during muscle activation, each panel experiences tensile stress between anode and cathode.

The 20 cm arm circumference serves as a lower bound for sleeve size along with a maximum panel length of 19 cm to allow clearance at the elbows and wrists. Given this area, three different flexible PV panel configurations are possible: one MPT3.6-150 panel (360 mW), two MPT3.6-75 panels (360 mW), or five SP3-37 panels (330 mW). Because the comparison is the PV power possible on the sleeve, the PV panel area is not exactly the same in each arrangement. The DC-DC converter mounts onto the sleeve and the Li-ion battery acting as the load fits within a pocket of the sleeve (Fig. 5); these components remain the same across all panel arrangements.

The sleeve with the attached PV panels is tested indoors (Fig. 6). The output power is shown in Fig. 6c and the efficiency of output power over input power is shown in Fig. 6d. The results show that the PV panel arrangement has a pronounced effect on output power. First, let’s compare the arrangements with one and two panels, where the panels have the same width (Fig. 2) and total PV area. In all test conditions, the single-panel arrangement yields a higher power and efficiency than the two-panel arrangement, indicating that it is the better choice between the two arrangements.

**Table 2:** PV Panel Specifications.

Panel	L	W	V$_{OC}$	V$_{MPP}$	Power
	(cm)	(cm)	(V)	(V)	(mW)
SP3-37	3.65	6.4	4.1	3.0	66
MPT3.6−150	14.6	7.4	4.9	3.6	360
MPT3.6−75	7.3	7.4	4.9	3.6	180

The arrangement with five PV panels consistently produces the highest power in all test conditions except for rotation angle $\theta = 0^\circ$ and tilt angle $\beta = 0^{\circ }$ (forearm directly faces the light). This result is despite its lower nominal power (330 mW vs 360 mW) yet larger panel area (117 cm$^2$ vs. 108 cm$^2$). In the zero tilt and zero rotation case, the power is less than 3 mW below the single-panel arrangement. When the rotation angle is $\theta =0^\circ$, the single-panel arrangement results in an equivalent or slightly higher efficiency than the five-panel arrangement. However, when the rotation angle is $\theta =45^\circ$, the five-panel arrangement consistently yields the highest output power and efficiency.

### Sleeve power production in various scenarios

Another metric in PV power generation is how much power the sleeve would produce in a variety of arm positions. The power produced from the panels and converters was measured on a mannequin posed in four different positions under direct sunlight that ranged from 770 to 820 Wm$^{-2}$ (Fig. 7). The average power recorded for three tests taken in each position, along with the power efficiency, is shown in Fig. 7.

The highest power produced was 93.9 mW, observed when the arm was straight down (mimicking standing) and the sleeve was receiving direct sunlight. Conversely, the lowest power was 13.5 mW, observed when the arm was up and angled at 90 $^\circ$ (mimicking talking on the phone); based on this position, the PV panels of the sleeve pointed slightly downward away from the direct sunlight. Over all the tests in different positions, the average power produced was 65 mW. As for efficiency of the system, the efficiency ranged from 55.9% to 81.9%, with an average value of 74.8%. The lowest efficiency occurred at the lowest input power, primarily due to the losses in the controller and gate driver that do not scale with power level. Although the efficiency can be further improved, these results verify output powers achieved for a wearable sleeve application when the arm was straight down (77.0 mW) and up (93.3 mW), and bent with the arm down (75.6 mW) and up (13.9 mW).

## Discussion

The primary concerns for evaluating PV performance in wearable devices are changes in power output (determined by both PV panel generation and converter efficiency) due to panel flexion and varying light angles. It was observed that MPP decreased during curvature. Thus, it is important to employ an active MPPT algorithm, such as P &O, to track these variations, ensuring that the panel continues to operate at its MPP.

Under normal activities, mechanical deformations that may be imposed on a sleeve-worn PV panel include strain between anode and cathode as a user flexes their forearm muscles and curvature as the panel conforms to a user’s arm. Strain along the panel length will be limited as a user’s forearm length is fixed. The panel responses to the tension characterization tests suggest that muscle flexion will have limited effect on the panel output. Little change in either output power or MPP voltage occurs as the panel moves from buckling to tension, with the biggest change as the panel receives full light intensity at the zero strain condition.

In contrast to tension, panel curvature and position does impact output, likely due to shading, decreased direct irradiance as the distance between panel and light source increases, and as the angle of incidence from a light source increases. The angle of rotation has a larger effect on the power production than the curvature of the panel, with limited shift on the MPP. These results are in agreement with previous work; Park et al.^36^ observed similar effects in a-Si panels, with a decrease in power as panel curvature increased due to smaller light intensity and panel self-shading, while the MPP voltage stayed relatively constant. The decreased power effect will be more pronounced as forearm size decreases, resulting from the higher curvature and larger variation in angle of incidence. However, the panel angle and position has a larger impact than curvature, so we expect that power output from a sleeve-based PV harvester would be generalizable across a population.

The tilt and rotation experiments also demonstrate the utility of a greater number of small panels over one large panel (Fig. 6). Under the special condition of no rotation or tilt, the single-panel (MPT 3.6-150) approach exceeded that of the five-panel (SP3-37), despite the 8% larger total PV area in the five-panel arrangement (Fig. 6b). The test fixture has a stepped curvature that simulates the shape of an arm, with lower curvature at its base (“elbow”) and higher curvature at its end (“wrist”). The single panel arrangement conforms poorly to the test fixture that results in a lower curvature over the panel, while the five-panel arrangement conforms well and has a larger curvature. At no rotation or tilt, the majority of the single panel has a high angle of incidence, while some portions of the panels are curved away from the light in the five-panel arrangement. When the test fixture is tilted or rotated, the light source still has a high angle of incidence to some part of the five-panel arrangement, while most of the single panel has a low angle of incidence. As a result, the disparity between single and five-panel arrangement performance increases as the tilt or rotation increases.

The two-panel (MPT 3.6-75) arrangement has the lowest performance of the three cases despite having the same PV area as the single panel. The behavior is again dominated by panel deformation. At no rotation or tilt, some portion of the panel is curved, resulting in lower performance than the single panel. Its performance is also worse than the five-panel case due to a smaller area. A second factor is the split between the two panels, which causes each of the two panels to conform to the curvature closer to the base. The change in curvature between the top and bottom of each panel is larger than in the five-panel configuration, resulting in the panel ‘bowing out’ relative to the surface of the test fixture and reducing its incident angle relative to the light source. Because the change in curvature for the single-panel case must be continuous (rather than a discontinuity present at the panel split), the overall curvature and conformality are lower, and the panel does not exhibit as much bowing out from the surface of the test fixture.

In these measurements, the smallest planar flexible panels can better conform to a desired body area, yielding higher user comfort and generating larger power under a range of expected conditions. A multi-panel strategy presents advantages between more generalized power generation, but adds complexity in fabrication and other form factor concerns, such as a need to remove more panels before washing the garment. As such, designers using PV panels should carefully consider the required power output as well as the broader use case of the garment.

The power measurements using the mannequin were taken in four different static positions and give a range of power expected during regular use in outdoor environments. The previous hypothetical example that draws 33 mW could be continually charged in the three more common forearm positions (down, up, and horizontal pointed up) which provided more than 75 mW. Even in the lowest-power position (horizontal pointed down), the measured output power was 13.9 mW, which is 42% of the nominal power draw and will proportionally lengthen the operating time from a single battery charge. Based on these power results and assuming the irradiance and resulting power could double in an outdoor condition, the battery should be larger than 51 mAh to limit the battery charging rate below 1C at 3.7V.

## Conclusion

Solar power is a promising energy source for providing supplemental power to wearable applications to reduce the required battery size or increase time between charges. However, there are challenges (e.g. power reduction) in moving from flat rigid panels to flexible panels that enable these applications. This work detailed the design process and considerations of a PV-powered wearable through a sleeve-based application and discussed its selection of PV panel and power converter. Tension applied to the PV panel (e.g. from muscle flexion) showed no clear effect on the panel’s output power, while increasing curvature of the panel reduced its output power and MPP voltage. Commercially-available flexible solar panels of different sizes and electrical characteristics were used to determine the best panel arrangement, while a boost converter that actively tracks the MPP was used in the power stage. The effect of body size on panel output was found to be lower than arm position; using a larger number of small panels can compensate for changing light angles more effectively than one large panel. The outdoor experimental results verify the power provided (65 mW on average) by flexible photovoltaic panels mounted on a sleeve to power a wearable device, even for forearm circumferences on the smaller end of the adult range (20.4 cm).

Due to their optimization for outdoor light, we did not demonstrate panel output under indoor lighting. However, we expect our findings of a higher panel number remaining more efficient under various body sizes and lighting conditions to generalize to indoor conditions. Future work will focus on 1) examining flexible PV panels intended for use in indoor environments to more broadly enable wearable devices under conditions where people will use them and 2) designing experiments that simulate a wide range of use conditions, including the role of reflected light from surrounding surfaces, garment construction, and possible strategies for panel washing or removal.

## Methods

### Experimental setup for tension, rotation, and curvature

To understand the effects of tension on expected power output, panels of each type (*N*=5) were placed in a universal materials characterization tester (5554, Instron) with a 250 W halogen lamp placed 19.5 cm from the center of the panel. The anode was anchored and the cathode position was controlled during the test. At the initial position, the panel was buckled outward towards the lamp. The final position in each test placed the panel in tension. The cathode position was increased by 1 mm when the panel was buckled and 0.1 mm when the panel was in tension and held while the IV sweep was measured with a DC programmable load (8542b, BK Precision). Each test was stopped when a tensile force greater than 20 N was reached.

To estimate the effect of arm circumference and rotation on power generation, panels were placed in a custom 3D printed test fixture with a circumference of 20 cm to 35 cm in 5 cm increments (Fig. 4b). These values correspond roughly with the 5*th* percentile for female arms to above the 95*th* percentile for male arms^45^. Three halogen lights (150 W each) were placed at a distance of 30 cm from the center of the panel, and the panels were manually rotated from alignment with the lamp at an angle of $\theta$. When the center of the panel directly faces the middle lamp, the rotation angle is $\theta =0^{\circ }$, and when the center of the panel rotates, the angle increases (Fig. 4b). The MPP of each panel was measured by sweeping the load voltage and measuring the output current with a DC programmable electronic load (8600, BK Precision). Five measurements were taken at each angle and curvature, and the irradiance in all cases was approximately 545 Wm$^{-2}$.

### Experimental setup for rotation and curvature with power converter

To determine the power harvested by the three different PV panel arrangements, the sleeve was mounted on a fixture that emulates a forearm, where the base of the test fixture is 35 cm in circumference and the top of the fixture (to simulate a wrist) is 20 cm in circumference. The sleeve with the attached PV panels is tested indoors (Fig. 6) with the three halogen lights stacked vertically. The sleeve fixture was rotated (defined as rotation angle $\theta$) and tilted (defined as tilt angle $\beta$), while the power at the output of the boost converter was measured. Measurements were taken at rotation angles of 0 $^\circ$ and 45 $^\circ$, and at tilt angles of 0 $^\circ$, 30 $^\circ$, 45 $^\circ$, and 60 $^\circ$. At each angle the measurement was taken two times, and the averages are reported. The three PV panel arrangements mounted on the test fixture are shown in Fig. 6b.

### Experimental setup for measurements in realistic positions

The arrangement with five PV panels was used in this experiment because it showed the highest power output in previous tests. The five-panel arrangement and boost converter system were placed on the forearm of a full-size mannequin, which had a maximum forearm circumference of 20.4 cm. The mannequin was placed in four static positions: (1) arm straight while pointing down, (2) arm straight while pointing up, (3) arm bent at 90 $^\circ$ while arm is down but forearm is angled upward, and 4) arm bent at 90 $^\circ$ while arm is down but forearm is angled downward (Fig. 7). In these different positions, the mannequin was placed outside in direct sunlight on a clear day and the irradiance was measured perpendicular to ground. The output power (at the output of the boost converter) was measured for each test over 30 s for three consecutive tests in each position.
